# Slipping Rib Syndrome in a Female Adult with Longstanding Intractable Upper Abdominal Pain

**DOI:** 10.1155/2018/7484560

**Published:** 2018-07-02

**Authors:** Noman Ahmed Jang Khan, Saba Waseem, Saad Ullah, Hassan Mehmood

**Affiliations:** Department of Internal Medicine, Conemaugh Memorial Medical Center, Temple University, Johnstown, PA, USA

## Abstract

Slipping rib syndrome is a rare cause of abdominal or lower chest pain that can remain undiagnosed for many years. Awareness among health care personnel of this rare but significant disorder is necessary for early recognition. Prompt treatment can avoid unnecessary testing, radiographic exposure, and years of debilitating pain. A 52-year-old female was evaluated for a 3-year history of recurrent abdominal and lower chest pain. Pain was sharp, primarily located in the lower chest and subcostal region left more than right, waxing and waning, nonradiating, and aggravates with specific movements. She underwent frequent physical therapies, treated with multiple muscle relaxants and analgesics with minimal improvement. Imaging modalities including CT scan, MRI, and X-rays performed on multiple occasions failed to signify any underlying abnormality. Complete physical examination was unremarkable except for positive hooking maneuver. Dynamic flow ultrasound of lower chest was performed which showed slipping of the lowest rib over the next lowest rib bilaterally left worse than right, findings consistent with slipping rib syndrome. Slipping rib syndrome is caused by hypermobility of the floating ribs (8 to 12) which are not connected to the sternum but attached to each other with ligaments. Diagnosis is mostly clinical, and radiographic tests are rarely necessary. Hooking maneuver is a simple clinical test to reproduce pain and can aid in the diagnosis. Reassurance and avoiding postures that worsen pain are usually helpful. In refractory cases, nerve block and surgical intervention may be required.

## 1. Introduction

Slipping rib syndrome is a rare cause of abdominal and lower chest pain that is often undiagnosed, overlooked, or misdiagnosed for many years. Many health care personnel are unaware of this rare entity, and failure of diagnosis may lead to unnecessary diagnostic interventions and extensive radiation exposure. We describe a case of a 52-year-old female who presented with 3 years of lower chest/upper abdominal pain and was later diagnosed as bilateral slipping rib syndrome.

## 2. Case Presentation

### 2.1. History

A 52-year-old female with no known past medical history was evaluated for a 3-year history of abdominal pain. Pain was sharp, primarily located in the lower chest and subcostal region left more than right, waxing and waning, nonradiating, and aggravates with certain nonspecific movements including forward lean. She was an accountant by profession and was never involved in any athletic activities. Her medications included over-the-counter acetaminophen and cyclobenzaprine. She underwent frequent physical therapy sessions and was treated with different analgesics with minimal improvement.

### 2.2. Physical Examination

Complete physical examination was unremarkable except for mild to moderate tenderness in the left more than the right subcostal area which was reproduced on hooking maneuver.

### 2.3. Diagnostic Workup

1. Prior to presentation, she underwent frequent imaging modalities on multiple occasions including CT chest, CT abdomen and pelvis, MRI abdomen and pelvis, and plain X-rays. All these modalities failed to identify any significant underlying abnormality.
2. EGD was also performed twice and was unremarkable on both occasions.
3. Dynamic flow ultrasound of the lower chest was performed to potentiate the diagnosis and revealed slipping of the lowest rib over the next lowest rib bilaterally left worse than right, findings consistent with bilateral slipping rib syndrome.

### 2.4. Course

Reassurance about the benign nature of disease was provided, and avoidance of pain-inciting postures was recommended. Her symptoms persisted despite conservative management, and intercostal nerve block was planned. Patient's symptoms remarkably improved with nerve block without requiring any surgical intervention.

## 3. Discussion

### 3.1. Introduction

Slipping rib syndrome also named as costal margin syndrome, clicking rib and rib-tip syndrome, was first identified in 1922 by Davies-Colley as a cause of severe abdominal pain due to overriding of the ninth and tenth rib [1]. The pathophysiology of slipping rib syndrome is uncertain.

Rib hypermobility caused by weakness of costochondral, sternocostal, or costovertebral ligaments is considered the primary underlying mechanism of slipping rib syndrome [2]. Holmes in 1943 proposed that pain in slipping rib syndrome is caused by recurrent subluxation of the costal margins of the 8th, 9th and 10th ribs making these ribs in close contact and cause irritation of the intercostal nerves.

### 3.2. Clinical Presentation and Diagnosis

**Figure 1:**
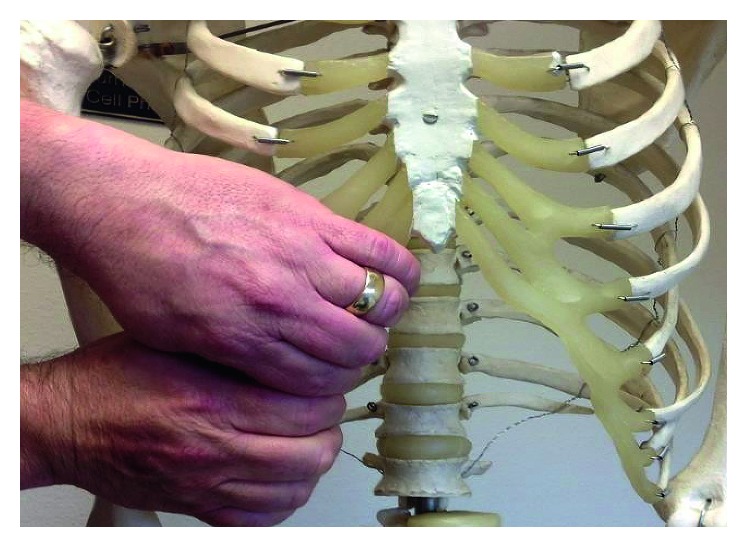
Hooking maneuver.

Slipping rib syndrome often presents as the lower chest, flank, or upper abdominal pain. It is usually a clinical diagnosis while imaging studies are often required to rule out other potential causes including rib fractures, costochondritis, and cholecystitis or hepatobiliary pathologies [2]. Pain is reproduced by performing hooking maneuver [3], first described in 1977. It is a simple test where the clinician places his fingers in the subcostal area and pulls in the anterior direction (Figure 1).

Pain or clicking indicates a positive test. The positive hooking maneuver test is usually followed by a nerve block. Relief of pain on the nerve block after positive hooking maneuver is highly suggestive of slipping rib syndrome. Dynamic flow ultrasound, as performed in our patient, is sometimes helpful in visualization of the ribs slipping against each other potentiating the diagnosis [4].

### 3.3. Management

Reassuring patients about the benign nature of this disease helps in relieving their anxiety and fear of having serious underlying illness. Reassurance and conservative measures are the key management options for patients having mild symptoms [5]. Avoidance of specific movements or postures triggering or aggravating pain is associated with favorable outcome [2, 6]. In more refractory and severe cases, nerve blocks should be performed. Our patient is currently being managed with nerve blocks repeated every 2 to 3 months. In very severe cases, resection of the slipping rib and accompanying cartilage is performed to alleviate symptoms [7].

### 3.4. Prognosis

The outcomes of both conservative and surgical management are quite favorable. Three case-series have been published explaining the satisfactory outcomes of simple reassurance, reassurance with local anesthetic injections, and surgical management, respectively [6, 8, 9].

## 4. Conclusion

Increased awareness of slipping rib syndrome as a potential cause of lower chest and abdominal pain can spare patients with years of pain and prevent unnecessary interventions and radiographic exposures. A simple clinical test like hooking maneuver is often enough to establish the diagnosis. Reassurance and avoidance of pain-triggering postures are often helpful. Refractory cases can be treated with nerve blocks and surgery.
